# For whom the bell tolls – do we overestimate wall enhancement of intracranial aneurysms?

**DOI:** 10.1007/s00330-023-10552-z

**Published:** 2024-01-19

**Authors:** Friedrich Götz

**Affiliations:** Institut für Diagnostische und Interventionelle Neuroradiologie, Medizinische Hochschule Hannover, Hannover, Germany

In this issue of *European Radiology*, a neurovascular team from the Netherlands presents interesting results from a longitudinal study of gadolinium-enhanced aneurysm wall imaging and risk of aneurysm growth and rupture [1]. Their findings are helpful for our understanding of unruptured intracranial aneurysms (UIAs).

Worldwide, more than 3% of the population without risk factors has an incidental intracranial aneurysm, but in China, 7% are reported [2]. Due to frequent use of CTA and MRA, the detection rate of UIAs is steadily increasing, and neurovascular centres have the impression that they are overrun with aneurysms. The question how to care best for these patients is complex and remains unanswered, at least in part.

An increasing number of scientific papers report on black blood imaging on the one hand and application of artificial intelligence in the detection and management of intracranial aneurysms on the other. Black blood imaging, which uses T1-weighted high-resolution 3D sequences and gadolinium injection, is a powerful method in the diagnosis of intracranial arteriosclerosis and vasculitis. There are several reports of aneurysm wall enhancement, both in ruptured and unruptured cohorts. Ruptured intracranial aneurysms commonly show wall enhancement. Studies affirm that wall enhancement is an ominous sign and might forecast rupture in incidentally detected aneurysms. Van der Kamp et al report that 37% of aneurysms have wall enhancement and 7% of these grow or rupture within two years [1]. Compared to aneurysms without wall enhancement, this means a 3.1 times higher risk of growth and rupture. The important finding of this research is that after adjustment for aneurysm size, risk halves and an increase of 1.4 times remains. This longitudinal observational study includes the largest population so far and challenges reports of aneurysm wall enhancement as an independent risk factor for rupture. Even if the results are not unexpected, validation by other groups is needed [3]. Limitations include a relatively small numbers of patients per centre, differences in hard- and software equipment, and evaluation of aneurysm wall enhancement by local imaging departments.

Pathophysiology of aneurysm wall enhancement is incompletely understood. There are concerns that aneurysm wall enhancement is not a simple reflection of histologic findings, but blood flow is contributing [3]. Histologic findings highlight the presence of cells and markers of inflammation along with vasa vasorum in the wall of ruptured and unruptured aneurysms. It is intelligible that imaging and histologic findings from relatively small cohorts do not fit like a key and a lock [3, 4]. Reproducibility and validation of aneurysm wall enhancement in longitudinal studies is pending. In vitro studies suggest that slow-flow phenomena can mimic aneurysm wall enhancement by generating a hyperintense signal adjacent to the aneurysm walls [5, 6]. Both, MSDE and DANTE prepared black blood sequences can reduce slow flow associated wall enhancement, but the first seems to be more practical in a clinical setting [5, 6]. Accumulated evidence contradicts the impression that wall enhancement is the only underlying process of aneurysm rupture. The time course of de novo development of aneurysms and their rupture rate is largely unknown and we do not consider it.

Many studies in *European Radiology* and other journals report on the development and validation of machine learning models for prediction of intracranial aneurysm rupture. Some studies focus on location, size, and morphology of aneurysms; others compare results of different models of logistic regression or PHASES. The number of aneurysms used for training and validation ranges from less than hundred to more than thousand. The most important shortcoming is that the longitudinal study design has not been applied so far and thus we have no correlation to the real world. One of the most promising approaches is the generation of a machine learning–based aneurysm rupture score, which combines an array of clinical data and several morphologic markers of aneurysm [7].

The type of UIA treatment has significant impact on health care costs. Missing aneurysm rupture due to abstaining from timely reparative therapy (preferably interventional) entails individual harm. In the study under discussion with a total follow-up duration was 994 aneurysm-years, 21 patients had 17 growing aneurysms without rupture (10/17 had wall enhancement) and five ruptured aneurysms (3/5 had wall enhancement). Two of the 21 patients with aneurysm growth or rupture had multiple aneurysms and the PHASES score was 6 compared to 4 in the stable group. A retrospective study of patients with aneurysmal subarachnoid haemorrhage calculated rupture risk; the authors point out that PHASES and UIATS are a weak tool for clinicians in the decision-making process [8]. If we opt for reparative therapy, we cannot ignore that treatment-related complications are in the magnitude of 3–10%. In this scenario, society must pay not only for non-essential therapy but also in addition for disability and care of the unluckiest.

Full of hope, we took aneurysm wall enhancement for an independent item to predict aneurysm rupture more precisely but the article of van der Kamp et al breaks the spell [1]. Prediction of UIA rupture risk warrants analysis of complex non-linear relationships of known and probably yet unknown significant variables. Without prospectively validated machine learning methods, we are relatively helpless when consulting with our UIA-patients. Soon, AI will outperform expert opinion of neurovascular specialists and intuition of doctors [7].

Already in 2008, Raymond et al pointed out our limited knowledge of the natural history of UIAs and stated that preventive therapy cannot be recommended [9]. In 2021, a Cochrane review “Treatments for unruptured intracranial aneurysms” concluded that the available studies are of very low methodological quality and the data provide little evidence of the best treatment [10]. At present, we can offer our patients no more than respectfully addressing their individual fears and needs.

## Declarations

### Guarantor

The scientific guarantor of this publication is Friedrich Götz.

### Statistics and biometry

No complex statistical methods were necessary for this paper.

### Methodology

• commentary
